# Differences in all-cause and death by suicide mortality between health care and other employees in Lithuania: a census-linked mortality follow-up study, 2011–19

**DOI:** 10.1093/eurpub/ckaf123

**Published:** 2025-07-21

**Authors:** Povilas Kavaliauskas, Domantas Jasilionis, Evaldas Kazlauskas, Giedre Smailyte

**Affiliations:** Department of Public Health, Institute of Health Sciences, Faculty of Medicine, Vilnius University, Vilnius, Lithuania; Laboratory of Cancer Epidemiology, National Cancer Institute, Vilnius, Lithuania; Max Planck Institute for Demographic Research, Rostock, Germany; Demographic Research Centre, Vytautas Magnus University, Kaunas, Lithuania; Center for Psychotraumatology, Institute of Psychology, Vilnius University, Vilnius, Lithuania; Department of Public Health, Institute of Health Sciences, Faculty of Medicine, Vilnius University, Vilnius, Lithuania; Laboratory of Cancer Epidemiology, National Cancer Institute, Vilnius, Lithuania

## Abstract

Lithuania has one of the highest adult mortality rates in Europe. A study analysing three large groups of healthcare employees, physicians, nurses, and assistant nurses, found no significant differences in all-cause mortality compared to other sectors. However, after controlling for education, physicians became the highest-risk group. Nurses and assistant nurses had the lowest risk, but no significant differences were found. Excess all-cause mortality of physicians after controlling for education is striking and needs to be investigated further. Given the low number of deaths by suicide among healthcare workers, more research is required to obtain more statistically robust inferences.

## Additional content

The author has now recorded their video: https://oup.cloud.panopto.eu/Panopto/Pages/Viewer.aspx?id=f8c3f085-b0d7-461c-934c-b31d011d21c5

## Introduction

Despite the improving situation, Lithuania still shows one of the highest all-cause and suicide rates among men in the European Union [1]. Excess suicide rates are related to significant inequalities by socioeconomic status and a high concentration of deaths by suicide in disadvantaged groups, such as unemployed and economically inactive men or those residing in small cities and rural areas [2]. More detailed evidence about certain risk groups is still missing. Medical workers such as nurses and physicians are subject to various occupational hazards, including work-related stressors (e.g. patient care, time pressure, administration). They are at increased risk of developing mental disorders [3]. Some studies from other countries report elevated suicide risk among certain healthcare occupations [4]. Such a disadvantage persists even though mortality from other causes among medical doctors in European countries has become generally lower than general population [5, 6]. The current study focuses on all-cause mortality and suicide risks among the three broad groups of health workers (nursing specialists, physicians, and other occupations).

## Methods

This study is based on the census-linked mortality dataset covering the entire population of Lithuania. The anonymized individual-level dataset includes all records from the 2011 Census and death and emigration records between 1 March 2011 (2011 Census date) and 31 December 2019. All record linkage procedures were implemented at Statistics Lithuania by its employees having permission to work with confidential data. Only anonymized data were provided for analysis based on the confidentiality of data provision rules by Statistics Lithuania. The current study uses the census and mortality follow-up data for those aged 25–50 years at the Census. The survival time is censored either at the moment of death or emigration. The final dataset includes 1 082 805 individuals of whom 34 427 were employed in the healthcare sector at the time of the 2011 Census. The primary analysis identified three groups: physicians, nurses, assistant nurses, and other health care employees (kinesitherapists, ergotherapists, etc.). Additionally, this study includes the categories of employed in all other sectors with high education, employed in all other sectors with lower education, all unemployed, and all economically inactive or people with unknown activity status. High educational status was identified using the self-reported information at the 2011 census and later classified using the International Standard Classification of Education 2011 by Statistics Lithuania. Deaths by suicide were identified by linking the 2011 Census data with the death records from the Population Register of Lithuania and Cause of Death Register at the Institute of Hygiene. The corresponding codes of the 10th revision of the International Classification of Diseases for death by suicide were X60 and X84 (intentional self-harm). The modelling results were reported using Cox regression mortality rate ratios (RR) and 95% confidence intervals (CIs). Statistical significance for mortality rate ratios was defined using *P*-values <.05. Permission for the study was obtained from the Vilnius Regional Bioethics Committee (ID: 2021/5-1350-826). The modelling was performed using STATA software (STATA Corp.).

## Results

During the follow-up period between 1 March 2011 and 31 December 2019, the whole study population experienced 32 900 deaths; 2902 of these deaths were identified as deaths by suicide. Three hundred and eighty-three deaths, including 16 deaths by suicide, occurred among healthcare employees. The deaths for three healthcare employee categories include 87 deaths and eight deaths by suicide for physicians, 168 deaths and three deaths by suicide for nurses and assistant nurses, and 128 deaths and five deaths by suicide for other healthcare employees.

The results presented in Table 1 (Model 1) indicate that physicians, nurses, and assistant nurses show the same all-cause mortality risk as other employees working in all other sectors. The same pattern was observed when comparing the three categories of healthcare employees. Meanwhile, suicide mortality shows a notably lower risk among the nurses and assistant nurses. After additional control for education (Model 2), physicians become the group with the highest all-cause mortality risk, even if compared to all other employees (Table 1, Model 2). Controlling for education almost does not change findings for suicide risk, except that rate ratios slightly decrease for nurses and assistant nurses (RR = 0.29, 95% CI 0.09–0.91). At the same time, controlling for education leads to an increasing tendency in suicide risk among physicians (RR = 1.62, 95% CI 0.81–3.28).

**Table 1.** Cox regression all-cause mortality and suicide mortality rate ratios and their 95% confidence intervals by occupational categories

Occupation	All causes		Deaths by suicide	
	Model 1	Model 2	Model 1	Model 2
1. Entire population^a^				
Employed in all other sectors (*D* = 12 800, *S* = 1286, *N* = 744 092)	1	1	1	1
Physicians (*D* = 87, *S* = 8, *N* = 7335)	0.87 (0.70–1.07)	1.31 (1.06–1.62)^b^	0.92 (0.46–1.84)	1.62 (0.81–3.28)
Nurses and assistant nurses (*D* = 168, *S* = 3, *N* = 15 793)	0.94 (0.80–1.09)	0.88 (0.76–1.03)	0.31 (0.10–0.97)^b^	0.29 (0.09–0.91)^b^
Other healthcare employees (*D* = 128, *S* = 5, *N* = 11 299)	0.97 (0.22–1.28)	1.04 (0.88–1.24)	0.53 (0.22–1.28)	0.60 (0.25–1.45)
2. Employed in health care only				
Physicians (*D* = 87, *S* = 8, *N* = 7335)	1	1	1	
Nurses and assistant nurses (*D* = 168, *S* = 3, *N* = 15 793)	1.11 (0.83–1.49)	0.88 (0.62–1.24)	0.22 (0.05–0.90)^b^	0.21 (0.04–1.17)
Other healthcare employees (*D* = 128, *S* = 5, *N* = 11 299)	1.14 (0.86–1.51)	0.94 (0.68–1.29)	0.48 (0.15–1.50)	0.47 (0.13–1.77)

Lithuanian population aged 25–50 years at the 2011 Census, 2011–19.

Model 1—controlled for age and sex; Model 2—controlled for age, sex, and education. *N*, number of participants at the 2011 Census baseline; *D*, number of all-cause and deaths by suicide; *S*, number of deaths by suicide.

a Mortality rate ratios for unemployed and other economically inactive are not shown.

b Statistically significant results, when *P* < 0.05.

## Discussion

This study demonstrated similar overall mortality risk for healthcare workers to the remaining occupational groups. The results of our study differ from other studies conducted in European countries, where overall mortality risk among physicians was consistently lower than in the general population [5–7]. Physicians and other medical workers are frequently early adopters of healthy behaviours based on their knowledge and economic resources. Prior studies also suggest that educational level and health behaviour might explain the lower mortality of medical workers compared to the general population. Other studies [5–7] show that doctors had lower mortality rates from smoking and lifestyle-related diseases such as lung cancer, cardiovascular diseases, and metabolic diseases. Meanwhile, our study found that controlling for education led to excess all-cause mortality risk among physicians.

Results on suicide risk among medical workers in our study are consistent with those previously reported in other countries [5–7]. Although we found an indication of increased suicide risk among physicians, the increase was not statistically significant. A higher risk of death by suicide in physicians was also reported in other studies. For example, a Norwegian study reported excess suicide rates among Norwegian medical physicians compared with other medical workers and the general population [5].

Suicidal behaviours are a complex multifactorial phenomenon, and our study design does not allow for a more detailed analysis of deaths by suicide among medical workers. Deaths by suicide can be associated with mental state, burnout or psychiatric disorder, among other factors. Occupational stress and burnout in medical workers may be related to an increased risk of death by suicide among physicians in comparison to the general population with higher education [8]. A study conducted in Lithuania showed that burnout was highly prevalent among anaesthetists and intensive care physicians, with two-fifths having high burnout levels. Moreover, burnout was strongly correlated with a problem with alcohol consumption, depression, cardiovascular and digestive disorders, use of sedatives, and overeating [9]. In addition, stigmatization of mental health problems can be a barrier to getting appropriate help through mental health services. Lastly, it is possible that death by suicide cases can be misclassified as other accidents [10].

Our study has some limitations. One of the concerns is the small number of deaths by suicide in some occupational groups. This limitation is an obstacle to obtaining statistically robust rate ratios and making reliable statistical inferences about the magnitude of the differentials. In addition, there is a lack of information about more specific risk factors such as mental disorders and alcohol abuse. Finally, we were not able to distinguish between full- and part-time employees.

A Lithuanian study reveals that healthcare workers face similar mortality risks to other occupational groups, but physicians face a significant excess of all-cause mortality. Additionally, suicide risk among physicians and other healthcare employees shows almost the same high risk as those employed in other sectors. These findings contribute to a better understanding of medical workers’ overall mortality and suicide risk, providing valuable information for well-being and preventive programmes.

## Data Availability

The original data used in this study were provided by Statistics Lithuania. Due to the agreement terms and data protection rules, these data cannot be passed to the third party and should be requested directly from Statistics Lithuania. The new findings suggest that physicians become the highest all-cause mortality risk group after controlling for education. Even if physicians and other healthcare employees do not exhibit a higher suicide risk, this finding should be considered worrying due to the very high suicide rate in the general population of Lithuania. More detailed data and research are needed to understand and address peculiar mortality patterns of healthcare employees in Lithuania, especially with respect to the mortality disadvantage of physicians. The findings should be an additional reason to strengthen prevention and policies to tackle general health problems and the mental health of healthcare employees in Lithuania.
